# Bone Health Index (BoneXpert) and parameters of peripheral quantitative computed tomography indicate overall adequate bone health in adolescents with chronic endocrine diseases at time of transition

**DOI:** 10.1371/journal.pone.0337842

**Published:** 2025-12-04

**Authors:** Sebastian Gippert, Markus Bettendorf, Johannes Hoos, Daniela Choukair

**Affiliations:** Heidelberg University, Medical Faculty Heidelberg, Department of Pediatrics I, Division of Paediatric Endocrinology and Diabetes, Heidelberg, Germany; UBC: The University of British Columbia, CANADA

## Abstract

**Background/Aim:**

Low bone mass is common in children and adolescents with chronic endocrine disorders. Peripheral quantitative computed tomography (pQCT) is used to quantify bone health. A new automated Bone Health Index (BHI) determination (BoneXpert^TM^) is available using digitized X-rays of the left hand taken for bone age (Greulich&Pyle, BA) determination. In this study, we determine the BHI in adolescents with various endocrine disorders and compare it to conventional pQCT indices to evaluate the BHI as a potential surrogate parameter for bone health and muscle strength in adolescents.

**Methods:**

134 adolescents (70 females) with chronic endocrine diseases and a median age of 17.6 years (IQR: 16.4,19.4) were included at the time of transition. Diagnoses were growth hormone deficiencies (GHD, n = 37), Turner syndrome (TS, n = 27), congenital adrenal hyperplasia (CAH, n = 22), small for gestational age (SGA, n = 20), multiple pituitary hormone deficiency (MPHD, n = 17) and Klinefelter syndrome (KS, n = 11). BA and BHI were assessed by BoneXpert™ (available for 104 patients) and were compared to a random subgroup with available pQCT parameters (n = 38: 13 CAH, 12 GHD, 7 TS and 6 SGA) assessed by XCT-2000 scanner. Further, grip strength was assessed by hand dynamometer (n = 134).

**Results:**

Median BHI-SDS for all patients available was −0.66 (IQR: −1.50,0.18, n = 104), lowest in patients with CAH (−1.04, IQR: −2.34,0.22, n = 22). The majority of patients (58.6%) had a BHI-SDS greater than −1, indicating adequate bone health. Median pQCT parameters and median grip strength showed a z-score greater than −1, suggesting an appropriate muscle strength for most patients. Patients with both available BHI-SDS and pQCT parameters (n = 32) showed significant positive correlations with several measurements: bone mineral content (65%) SDS (ρ = 0.576, p < 0.001); metaphyseal (4%) site total BMD SDS (ρ = 0.492, p = 0.005); total cross-sectional area SDS (ρ = 0.379, p < 0.032) and SSI SDS (ρ = 0.417, p = 0.018). In addition, BHI-SDS correlated positively with muscle cross-sectional area SDS (ρ = 0.352, p = 0.066, n = 32) and grip strength SDS (ρ = 0.205, p = 0.050, n = 104).

**Conclusions:**

At the time of transition, the bone health of adolescents with chronic endocrine diseases is mostly appropriate, as estimated by BoneXpert^TM^ and pQCT. Grip strength was also adequate in the majority of patients. In addition, this study is the first to demonstrate a significant correlation between these methods in adolescents. Consequently, the BHI has the potential to be an effective screening tool for assessing bone health, characterized by wide availability and low radiation exposure, though additional analyses are needed.

## Introduction

Bone health and the lifetime risk of osteoporosis are important issues for adolescent patients with chronic endocrine diseases, as peak bone mass — the maximum amount of bone accrued during young adulthood — is influenced during puberty and adolescence [1]. Any interruption to normal physiological processes during this time, caused by illness or other factors, may result in a reduction in eventual peak bone mass. Although endocrine disorders such as Ullrich-Turner syndrome, congenital adrenal hyperplasia and growth hormone deficiency have different underlying causes, they can all negatively affect bone metabolism and thus prevent the development of peak bone mass. Bone density measurements during the transition to adult healthcare provide an opportunity to evaluate the long-term effectiveness of therapy and to compare different patient groups, in order to identify those that require special attention with regard to bone health. However, assessing bone health in children and adolescents is difficult. In addition to clinical information and biochemical surrogate parameters of skeletal health, a variety of radiographic techniques can be used [2]. Bone mineral density (BMD) is mainly assessed by dual-energy X-ray absorptiometry (DXA) [3,4], as peripheral quantitative computed tomography (pQCT) [3,5,6] is a three-dimensional technique that uses the attenuation of x-rays to build bone images. Cortical and trabecular bone compartments vary in density, and the differential attenuation of x-ray beams in the three-dimensional reconstruction allows for separate determination of trabecular and cortical BMD [1]. DXA only measures areal BMD, neglecting the possibility of reduced bone size in children with smaller body size [7]. In contrast, pQCT can discriminate between size and mass effects [8,9], but this method is not widely available and requires expertise to interpret the results. pQCT is time-consuming, costly and also involves exposure to radiation (<1 µSv) [10].

The measurement of the cortical thickness in the metacarpals from plain radiographs has been a standard method of assessing skeletal health/mineralization [11]. Since the 1950s, X-rays of the left hand have been widely used in children, mainly for bone age determination according to Greulich and Pyle [12] and Tanner and Whitehouse [13]. Both methods require experienced interpreters to determine skeletal age and are subject to considerable intra- and inter-observer variability [14]. Recently, a fully automated image analysis method, BoneXpert^TM^, has been developed to determine bone age from a digital hand X-ray [15]. Further, the software determines the Bone Health Index (BHI), which measures bone mass based on the cortical thickness of the three middle metacarpals and their width and length [16]. The BHI is referenced as a function of bone age, not chronological age, to compensate for the considerable variability in size, proportions, and maturation in growing children during different stages of development [16]. The software automatically calculates standard deviation scores (SDS values) for the BHI measurements, that are based on values from Caucasian children and adults [16–18]. To date, the BHI has been validated in healthy children and adults [16–19].

Besides bone mass, the muscular apparatus is a significant point of interest, given that bone strength adapts to mechanical loads, which are primarily created by muscle force [8,20]. pQCT data, e.g., the cross-sectional area of the muscles, but also grip strength can provide insight into this interaction [21].

The aim of this study was to investigate bone health assessed by BHI measurements using the automated BoneXpert^TM^ method in patients with chronic endocrine diseases at the time of transition from paediatric to adult care. To substantiate these findings, the BHI measurements were compared to the established pQCT method and related to muscle strength.

## Materials and methods

The study was designed as a prospective, single-centre and noncontrolled study. It was approved by the Ethics Committee of the University Medical Faculty Heidelberg (S-019/2011). Before participation, written informed consent was obtained from patients and/or their respective guardians. All investigations were implemented in a standardized routine transition examination and analysed retrospectively in accordance with the ethical standards of the institutional and national research committee, as well as the tenets of the 1964 Helsinki Declaration and its subsequent 2013 amendments, or equivalent ethical standards.

### Patient characteristics

A continuously enrolled cohort of 134 adolescents (70 females and 64 males) were followed in the Division of Paediatric Endocrinology at the Children's University Hospital Heidelberg between 29^th^ June 2011 and 03^rd^ August 2016. Patients with different endocrine diseases were included: multiple pituitary hormone deficiencies (n = 17, MPHD), isolated organic and idiopathic growth hormone deficiency (n = 37, GHD), congenital adrenal hyperplasia (n = 22, CAH), Turner syndrome (n = 27, TS) short stature after being born small for gestational age (n = 20, SGA) and Klinefelter syndrome (n = 11, KS) (Table 1). Diagnostic procedures and treatment of this cohort were performed according to German or international standards, as previously described [22,23]. The criteria for including patients were (i) having a chronic endocrine disease and (ii) confirming full pubertal development and reaching near-final height. Confirmation was done with reaching a skeletal age more than 16 years in boys and a change in their voice and more than 14 years in girls and having menarche [24]. According to the table of Bayley and Pinneau at this time 98% of their final height is reached [25].

**Table 1:** Patient characteristics.

Diagnosis	Total	CAH	GHD	SGA	MPHD	TS	KS
**Biological sex (f/m)**	134 (70/64)	22 (12/10)	37 (14/23)	20 (10/10)	17 (7/10)	27 (27/-)	11 (-/11)
**CA (years)**	17.6 (16.4, 19.4)	18.2 (16.9, 22.4)	17.1^§^ (16.0, 18.8)	15.8* (14.6, 16.8)	20.9^&^ (17.5, 26.0)	17.7^+^ (16.7, 20.2)	18.3 (17.8, 18.6)
**BA (years)**	16.6 (15.8, 17.4)	16.9 (16.2, 17.6)	16.3 (15.0, 17.2)	16.1 (14.66, 16.8)	17.3 (16.2, 17.6)	17.3^++^ (16.4, 17.7)	17.0 (16.1, 17.7)
**Difference CA-BA**	−0.6 (−2.4, 0.2)	1.7 (−0.3, 6.0)	1.2 (−0.1, 2.0)	0.0** (−0.6, 0.3)	3.8^&&^ (0.6, 4.9)	0.5^+^ (−0.3, 3.3)	1.5 (−0.3, 2.0)
**Near Final Height (NFH) SDS**	−1.31 (−2.46, −0.41)	−0.92 (−1.86, −0.28)	−1.32 (−1.95, −0.40)	−1.86*** (−2.69, −1.21)	−0.63 (−2.43, 0.32)	−2.70^+++^ (−3.55, −1.70)	0.61^%^ (0.26, 1.58)
**Target Height (TH) SDS**	−0.73 (−1.40, −0.06)	−0.61 (−1.63, −0.02)	−0.77 (−1.51, −0.18)	−1.06 (−1.92, −0.23)	−0.39 (−1.20, 0.37)	−0.78 (−1.18, 0.1)	−0.37 (−0.92, 0.25)
**Difference NFH – TH**	−0.49 (−1.49, 0.24)	−0.30 (−1.43, 0.39)	−0.20 (−0.97, 0.32)	−0.57 (−1.16, −0.20)	−0.51 (−1.19, 0.56)	−1.87^++++^ (−2.55, −1.26)	1.30^%%^ (0.64, 1.96)
**BMI SDS**	0.26 (−0.52, 1.31)	0.36 (−0.35, 1.71)	−0.07 (−1.56, 0.55)	−0.32**** (−1.07, 0.98)	1.47^&&&^ (0.11, 2.44)	0.59 (−0.04, 1.45)	0.34 (−0.52, 1.32)

*CAH* congenital adrenal hyperplasia; *GHD* isolated growth hormone deficiency; *SGA* small for gestational age; *MPHD* multiple pituitary hormone deficiency; *TS* Turner syndrome; *KS* Klinefelter syndrome; *f* female; *m* male; *CA* chronological age; *BA* bone age according to Greulich&Pyle; Data are given as median and IQR. Statistics by Kruskal-Wallis-Test: *p < 0.05 vs. all other groups; ^§^p < 0.05 vs. MPHD and KS; ** p < 0.05 vs. GHD and MPHD, ^+^ p < 0.05 vs. MPHD; ^&^ p < 0.05 vs TS, GHD and SGA; ^%^ p < 0.05 vs all other groups, ^++^ p < 0.05 vs SGA and GHD, ^&&^ p < 0.05 vs TS and SGA ^+++^ p < 0.05 vs GHD, CAH, KS and MPHD, *** p < 0.05 vs. GHD, CAH and KS, ^++++^ p < 0.05 vs all other groups, ^%%^ p < 0.05 vs SGA, MPHD, GHD and CAH, ^&&&^ p < 0.05 vs SGA, GHD, CAH and KS, **** p < 0.05 vs MPHD and TS.

In 104 patients, BHI was available. As the contract for pQCT examinations expired there were no longer pQCT available in our institution. Therefore, we could analyse the pQCT data of only 38 patients (S1 Table).

All patients with CAH were diagnosed before newborn screening was established in 1999 in Germany. Nine patients were diagnosed postnatal, four patients in the first year of life, and nine patients prepubertal. We classified fifteen patients as salt wasting (SW), two as simple virilizing (SV), and five as non-classical CAH. Virilized female neonates were classified according to the Prader scale, and five of them with Prader stage II and higher underwent genitoplasty. At the time of transition, 10 patients received dexamethasone (0.25–0.75 mg/d in the evening) and 0.05 mg fludrocortisone twice daily, four patients took hydrocortisone (15 mg/m^2^/d in three doses) together with 0.05 mg fludrocortisone twice daily, and one patient was treated with the combination of 20 mg hydrocortisone in the morning, 3 mg prednisolone in the evening, and 0.05 mg fludrocortisone twice daily. Two patients received only hydrocortisone (15 mg/m^2^/d in three doses) or five patients only dexamethasone (0.25–0.75 mg/d once in the evening).

Out of 54 patients with GHD or MPHD, 52 received GH (0.025 mg/kg/day in one dose subcutaneously) after their diagnosis was confirmed in childhood using standard arginine and insulin tolerance tests. Five patients with MPHD and two with GHD were still receiving GH at the time of transition. Other treatments included L-thyroxine (25–200 μg once in the morning, n = 23/54) for central hypothyroidism, hydrocortisone (5–20 mg three times per day; n = 13/54) for central adrenal insufficiency, and either estradiol valerate (2 mg in one dose daily) in combination with chlormadinonacetate CMA (n = 5; from day 1 to day 12 each month), testosterone undecanoate (250 mg/month or 1000 mg very three months by intramuscular injection; n = 6) or human chorionic gonadotropin (500–1500 IU twice a week by subcutaneously injection; n = 1) for hypogonadotropic hypogonadism (HH). Twenty five of 27 patients with TS received previously GH (0.045–0.05 mg/kg/day by subcutaneous injections), while two patients remained untreated due to lack of growth potential at diagnosis. Twenty patients received estradiol valerate (2 mg in one dose) in combination with cyclic CMA (2 mg; from day 1 to day 12 every month). Six girls with TS (X mosaicism: 3:45, X0/46, XX, and 3:45, X0/47, XXX) developed spontaneous regular menses. Six patients received L-thyroxine (25–125 μg once in the morning) due to Hashimoto’s thyroiditis. One patient with KS was treated with transdermal testosterone 2.5 g/daily, two with testosterone undecanoate 250 mg/month injected intramuscularly and seven patients received testosterone undecanoate 1000 mg very three months by intramuscular injection. Due to adequate endogenous testosterone production, one patient remained untreated. Twenty patients with SGA were treated with GH (0.035 mg/kg/day subcutaneously).

### Examinations

During a standard examination for children at the time of transition, several parameters were measured: chronological age (CA, years), near-final height (NFH, cm, using a wall-mounted Harpenden stadiometer (Mentone Educational, Moorabbin, VIC, Australia)), weight (kg, using an electronic scale (Seca, Hamburg, Germany)), body mass index (BMI, kg/m^2^) and grip strength (GS, Newton, N, mean of three measurements for the non-dominant hand with a handheld Jamar dynamometer (Sammons/Preston, Jackson, MI, USA)). Tanner's calculation of the midparental height was used to determine the target height [26]. The results were converted to standard deviation scores (SDS) using the appropriate German references [27–31]. Fracture data was analysed retrospectively via the hospital´s electronic medical system.

### Bone Health Index (BHI)

Conventional a.p. radiographs of the left hand were taken to assess bone age. The images were then converted to DICOM (digital imaging and communications in medicine) format. BA, BHI, and BHI-SDS were calculated using BoneXpert^TM^ software (BoneXpert^TM^ version 2, Visiana, Holte, Denmark) as earlier published by Thodberg et al. [16,22,32]. The computer software determines the BHI by measuring the cortical thickness (T), length (L) and width (W) of the three middle metacarpal bones II–IV. The BHI is then calculated and defined using the formula BHI = π T (1 – T/W)/(LW)∘0.33 according to the protocol of Visiana, Hørsholm, Denmark. The measured BHI is converted to SDS based on a reference population of healthy children and adults of identical bone age and analysed as a function of bone age [16,18]. According to the manufacturer's data, the coefficient variant (CV%) for BHI is 1.4% at age 10 [33]. According to the official position of the International Society for Densitometry, a pediatric definition for low bone mass does not exist and should not be used [34]. In our study, we examine adolescents transitioning to adult medicine, so we use the WHO guidelines for adults to define a T-score > −1 as normal and transfer this definition to BHI-SDS [35].

### Peripheral quantitative computed tomography (pQCT)

The distal end of the radius (4% site) and the upper part of the shaft (65% site) of the non-dominant forearm were examined using pQCT (XCT-2000 scanner, Stratec Inc., Pforzheim, Germany), as explained in detail before [36]. Briefly, pQCT analysis was performed at the nondominant forearm using a technology (XCT 2000, Stratec, Inc., Pforzheim, Germany) described previously [10]. The scanner was positioned on the distal and proximal forearm respectively and a coronal computed radiograph (scout view) was carried out. The scout view was used to position the scanner at the measurement site whose distance from the ulnar styloid process corresponded to 4% and 65% respectively, of forearm length. A single tomographic slice of 2.0 mm thickness was taken at a voxel size of 0.4 mm. Image processing and calculation of numerical values were performed using the manufacturer’s software package (version 5.40, Stratec, Inc.) [37,38]. There was no evaluation of motion artefacts in the pQCT images. Total and trabecular BMD were calculated using the software provided by the manufacturer at the metaphyseal site (4%). Total BMD is defined as the mean mineral density of the total cross-section [21]. At the diaphyseal site (65%), cortical cross-sectional area was determined by detecting the outer and inner cortical bone contour at a threshold of 710 mg/cm^3^. Peripheral areas at the outer edges of the bone with a density between 20 and 60 mg/cm³ were interpreted as representing muscle. Cortical cross-sectional area, total BMD, cortical BMD, and muscle cross-sectional area were calculated by the manufacturer’s software. Cortical thickness is derived from these primary measures as formerly described [37]. The Strength–Strain Index (SSI) measures how strong a bone is when twisted or bent, and it is determined by multiplying the section modulus by the volumetric cortical BMD, adjusted to the highest normal cortical BMD in human bones [39]. It reflects a good approximation of the mechanical strength of human bone [9,39]. The pQCT and dynamometer results were compared with those of a German reference population from the DONALD study using identical methodology [37,38,40]. Results in these patients were converted into sex- and height age-specific z-scores to consider the reduced body size of the patients. Following formula was used: z- score = [(test result for a patient) – (height age-specific mean in reference population)]/ (height age-specific SD in reference population) [37,38].

### Statistical analysis

Unless otherwise indicated, data are given as median and interquartile ranges (IQR). According to the literature, standardized data of pQCT measurements are referred as z-scores, standardized data of BHI as SDS. The Kolmogorov-Smirnov test was used to determine the non-normality of all the data. The nonparametric Kruskal-Wallis test with post-hoc analyses (Bonferroni test) were used for comparison of groups, and correlation coefficients were calculated according to Spearman. The Spearman coefficients were interpreted according to Cohen: weak correlation |ρ| >=.10, moderate correlation |ρ| >=.30 strong correlation |ρ| >=.50 [41]. P < 0.05 was considered statistically significant. We provide the overall bias and limits of agreement (Bland-Altman-plots) and we examined proportional bias using linear regression analysis, regressing the difference between the methods on their mean. Since the study was an exploratory analysis, no adjustment for multiple testing was done. SPSS Statistics 28.0.1.0 (IBM, Armonk, New York, USA) was used for statistical analysis.

## Results

134 patients were included in this study (n = 70, 52.2% female). The median chronological age at examination was 17.6 years (IQR: 16.4,19.4). Patients with SGA were significantly younger than patients in all other groups (p < 0.001). Median bone age was retarded by −0.58 years (IQR: −2.42,0.24). In general, the difference between chronological age and bone age was not significant, except for patients with MPHD, whose bone age was retarded by three years (p < 0.05). Five of 134 patients had a fracture in their life (3,7%): 2 fractures of the long bones (humerus), 1 lumbar spine fracture, 1 fracture of the hand, 1 fracture of a finger. This results in a prevalence of 0,12% per year.

Patient characteristics are given in detail in Table 1. Target height showed no significant difference between all groups (p = 0.760). Patients with SGA and TS showed a significantly lower near-final height than all other groups (p < 0.05) and KS patients had a higher near-final height than all other groups (p < 0.05). SGA patients reached their target height (difference NFH-TH: −0.57 SDS, IQR: −1.16, −0.20 SDS), whereas patients with TS showed a significantly larger difference (−1.87 SDS, IQR: −2.55, −1.26 SDS, p < 0.05). Patients with KS were the only patient group who exceeded their individual target heights (1.30 SDS, IQR: 0.61, 1.96 SDS, p < 0.05).

### Bone health index

**Fig 1:**
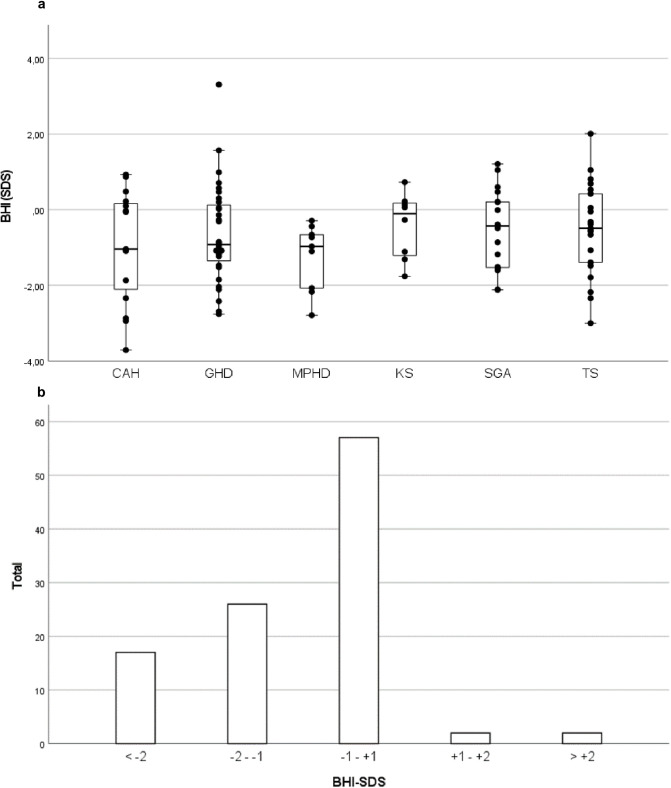
BHI-SDS for all patient groups, n = 104 (CAH: n = 15; GHD: n = 32; MPHD: n = 9; KS: n = 8, SGA: n = 19, TS: n = 21) and prevalence distribution. Differences between diagnoses were not significant (p = 0.634). BHI-SDS: Bone Health Index-standard deviation score; CAH: congenital adrenal hyperplasia; GHD: growth hormone deficiency; MPHD: multiple pituitary hormone deficiencies; KS: Klinefelter syndrome; SGA: small for gestational age; TS: Turner syndrome.

The median BHI-SDS for all patients available (n = 104) was −0.66 (IQR: −1.50, 0.18), the lowest in patients with CAH (−1.04, IQR: −2.34, 0.22), the highest in patients with KS (−0.11, IQR: −1.26, 0.20) (Fig 1a). However, the differences between the groups were not statistically significant (p = 0.634). Female patients had a slightly higher BHI-SDS (−0.56, IQR: −1.49, 0.26) than males (−0.86, IQR: 1.57, 0.15) (p = 0.458). The majority of patients (n = 61/104, 58.6%) had a BHI-SDS greater than −1, indicating unimpaired bone health (Fig 1b). BHI-SDS lower than −1 SDS were observed in 53.3% of patients with CAH, 43.8% of patients with GHD, 36.8% of patients with SGA, 44.4% of patients with MPHD, 33.3% of patients with TS, and 37.5% of patients with KS. In our cohort, no significant hydrocortisone effect could be demonstrated (p = 0.771). There was no significant difference in BHI-SDS between patients with a history of fractures (−1.29; IQR: −2.16, −0.55) and without (−0.66; IQR: −1.48, 0.20) (p = 0.638).”

### pQCT indices and grip strength

Thirty-eight patients (22 males) were available for pQCT analyses (see S1 Table). Two patients have previously been documented as having fractures (1.5%). Table 2 shows the bone density parameters, which were measured by pQCT at the proximal and distal radius and converted to z-scores. All median parameters regarding the bone density showed a z-score greater than −1, suggesting an appropriate BMD. The median z-scores for bone geometry and bone-muscle relationship also showed no significant impairment (defined as <−1). However, there is a slight reduction in cortical thickness, muscle cross-sectional area, and grip strength. For details, see Table 3.

**Table 2:** Bone density parameter z-scores at the distal radius [metaphyseal (4%) site] and the proximal radius [diaphyseal (65%) site] measured by peripheral quantitative computed tomography.

Parameter	All patients n = 38	Female n = 22	Male n = 16
Total bone mineral density_4%_ (SDS)	0.95 (−2.06, 0.07)	0.16 (−0.96, 1.38)	0.86 (−0.32, 2.01)
Trabecular bone mineral density_4%_ (SDS)	−0.41 (−1.51, 0.31)	−0.99 (−1.54, 0.08)	−0.07 (−1.46, 0.69)
Total bone mineral density_65%_ (SDS)	0.13 (−1.03, 1.00)	0.13 (−0.91, 1.19)	0.01 (−1.06, 0.92)
Cortical bone mineral density_65%_ (SDS)	1.28 (0.03, 2.55)	1.90 (0.28, 2.73)	0.79 (−0.01, 1.67)
Bone mineral content_65%_ (SDS)	1.20 (0.66, 2.01)	1.20 (0.70, 1.75)	1.52 (0.47, 3.54)

Data are given as median and IQR.

**Table 3:** Z-scores of the bone geometry and strength of the proximal (65%) radius measured by peripheral quantitative computed tomography and parameters of the functional muscle-bone relationship.

Parameter	All patients n = 38	Female n = 22	Male n = 16
Cortical thickness (SDS)	−0.84 (−1.74, −0.14)	−0.81 (−1.20, −0.25)	−1.08 (−1.97, −0.07)
Total cross-sectional area (SDS)	1.25 (0.24, 2.73)	1.23 (0.34, 1.58)	1.78 (0.06, 4.23)
Strength-Strain Index (SDS)	−0.26 (−0.99, 0.73)	**−**0.26 (−0.99, 0.52)	−0.05 (−1.42, 1.65)
Muscle cross sectional area (SDS)	−0.85 (−1.53, 0.18)	**−**0.89 (−1.46, 0.27)	−0.85 (−1.61, 1.14)
Grip strength (SDS) *(n = 116)*	−0.90 (−1.55, 0.23)	−0.91 (−1.53, 0.29) n = 64	−0.90 (−1.88, 0.18) n = 52

Data are given as median and IQR.

### Comparison of BHI with pQCT indices

**Fig 2:**
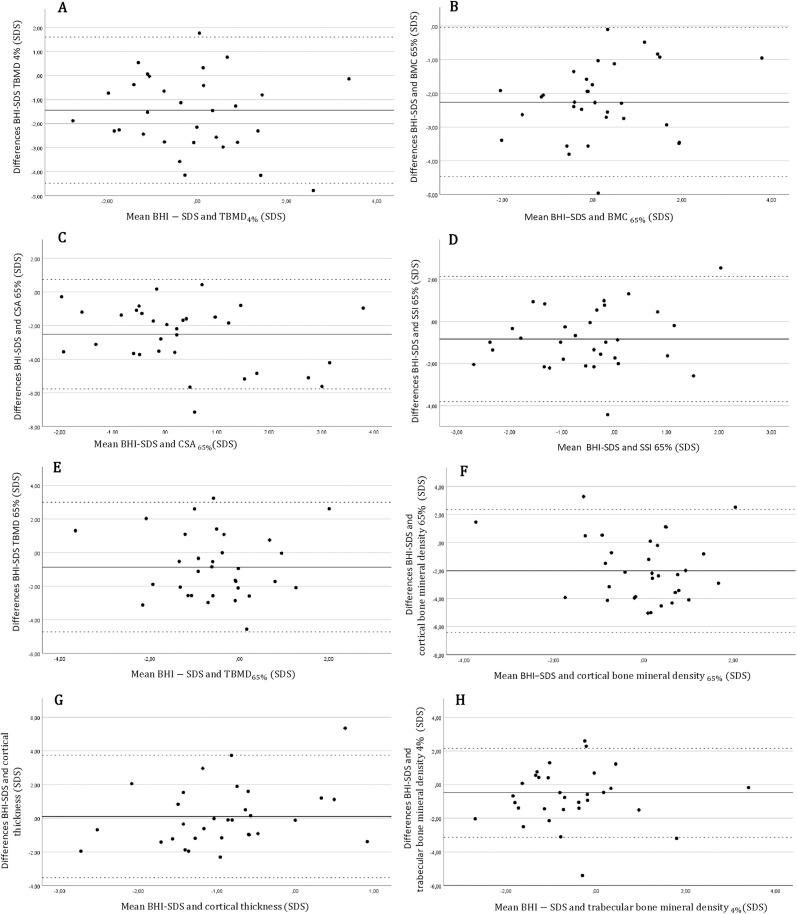
Bland-Altman-plots of BHI-SDS and pQCT readings (n = 32). A: Total bone mineral density _(4%)_ (SDS); B: Bone mineral content _(65%)_ (SDS); C: Total cross-sectional area _(65%)_ (SDS); D: Strength-Strain Index _(65%)_ (SDS); E: Trabecular bone mineral density _(65%)_ (SDS); F: Cortical bone mineral density (SDS); G: Cortical thickness (SDS); H: Trabecular bone mineral density (_4%)_ (SDS). *BHI: Bone Health Index*; *pQCT*: *peripheral quantitative computed tomography.*

A strong correlation was found between BHI-SDS and bone mineral content _(65%)_ SDS (ρ = 0.576, p < 0.001). There were moderate correlations between BHI-SDS and total BMD SDS at the metaphyseal _(4%)_ site (ρ = 0.492, p = 0.005) and between BHI-SDS and total cross-sectional area _(65%)_ SDS (ρ = 0.379, p = 0.032). SSI _(65%)_ SDS also showed a moderate correlation coefficient (ρ = 0.417, p = 0.018). All Spearman correlations are shown in Table 4. Fig 2 shows the Bland–Altman plots, which display that there is no trend in the difference depending on the mean in general. This suggests that the two methods are reasonably close, with nearly all observations within the limits of agreement. Though the mean bias for BHI-SDS and certain parameters (bone mineral content (65%) SDS, total cross-sectional area (65%) SDS and bone mineral density SDS) was about −2 SDS, indicating a systematic underestimation bias. Linear regression analyses showed no proportional bias for all parameters (S3 Table).

**Table 4:** Spearman correlations of BHI SDS and pQCT values.

Parameter	Spearman coefficient ρ	95%-CI	Significance p
Total bone mineral density_65%_ (SDS) (n = 32)	0.044	−0.319, 0.396	0.811
Cortical bone mineral density_65%_ (SDS) (n = 32)	−0.136	−0.471, 0.233	0.457
**Bone mineral content**_**65%**_ **(SDS) (n = 32)**	**0.576**	**0.275, 0.775**	**<0.001**
**Total bone mineral** **density**_**4%**_ **(SDS) (n = 31)**	**0.492**	**0.157, 0.726**	**0.005**
Trabecular bone mineral density_4%_ (SDS) (n = 31)	0.191	−0.185, 0.519	0.302
Cortical thickness (SDS) (n = 31)	−0.031	−0.391, 0.336	0.867
**Total cross-sectional area (SDS) (n = 32)**	**0.379**	**0.024, 0.649**	**0.032**
**Strength-Strain Index (SDS) (n = 32)**	**0.417**	**0.069, 0.674**	**0.018**
Muscle cross sectional area (SDS) (n = 28)	0.352	−0.036, 0.648	0.066
**Grip strength (SDS)** ***(n = 116)***	**0.349**	**−0.010, 0.629**	**0.050**

Data are given as median and IQR, all parameters are height adjusted. Significant correlations are highlighted (bold).

### Comparison of BHI (BoneXpert^TM^) with functional bone-muscle relationship

**Fig 3:**
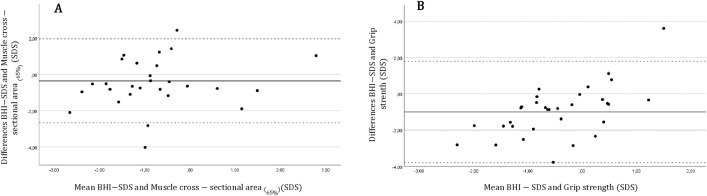
Bland-Altman-plots of BHI-SDS and parameters of functional bone-muscle relationship. A: Muscle cross sectional area _(65%)_ (SDS), n = 32, ρ = 0.352, p = 0.066; B: Grip strength _(65%)_ (SDS), n = 104, ρ = 0.205, p = 0.050. *BHI: Bone Health Index.*

There were moderate positive correlations between BHI-SDS and muscle cross-sectional area _(65%)_ SDS (ρ = 0.352, p = 0.066) and between BHI-SDS and grip strength SDS (ρ = 0.205, p = 0.050). Bland-Altman-plots are shown in Fig 3. However, linear regression analyses of the differences and the means showed a proportional bias for grip strength SDS and BHI-SDS (B = 0.962, p < 0.001).

## Discussion

To our knowledge, this is the first study reporting and comparing BHI-SDS for different rare endocrine diseases during the period of transition, validated by pQCT measurements.

So far, BHI-SDS has been studied in different groups of patients. For instant, it was found to be lower in patients with KS [42], somewhat lower in patients with juvenile idiopathic arthritis [43], Marfan syndrome [44], isolated growth hormone deficiency [45] before GH treatment but not after, and in patients with diabetes mellitus type 1 [46]. The BHI-SDS was also found to be lower in children with chronic kidney disease [47], multiple pituitary hormone deficiency [45], and Fontan circulation [48]. In addition, a severe reduction in BHI-SDS was observed in children with intestinal failure [49].

We observed a reduced BHI-SDS (−0.66; IQR: −1.50, 0.18) in the entire study population, with the lowest levels observed in patients with congenital adrenal hyperplasia. Children and adolescents with CAH are exposed to fluctuating cortisol and androgen levels [50]. As demonstrated by Halper et al., there was reduced bone mineral density in children with CAH; however, the correlation with hydrocortisone doses was inconclusive [50,51]. In our cohort, no significant hydrocortisone effect could be demonstrated (p = 0.771).

In addition, BHI-SDS was lower in patients with multiple hypopituitarism and isolated growth hormone deficiency. GH therapy is known to have additional beneficial effects on lean body mass gain and bone mineralization. Therefore, GH treatment should be continued until peak bone mass is reached [52]. Before starting growth hormone therapy, the median BHI-SDS was lower in patients with isolated growth hormone deficiency and multiple pituitary hormone deficiencies, at −0.97 and −1.85, respectively [45]. Significant increases in BHI-SDS were observed in all patients with growth hormone therapy; however, patients with lower pre-treatment BHI-SDS remained significantly lower [45].

We demonstrate a concordance between the BHI results and the pQCT data by validating the BHI results with pQCT measurements. The BHI-SDS had strong positive correlations with the total bone mineral density SDS, bone mineral content SDS, and total cross-sectional area SDS. These results were supported by Schündeln et al., who also found a significant positive correlation between total BMD measured by pQCT and BHI (r = 0.39; p < 0.0001) in patients with endocrine or hematologic diseases [2]. Though the Bland-Altman plots of these parameters showed a systematic underestimation, so they should be interpreted with caution. Trabecular structures are not adequately reflected by the BHI, as other authors and we have not been able to demonstrate a strong correlation between trabecular bone mass measured by pQCT and the BHI-SDS [2]. Furthermore, to investigate trabecular bone, lumbar spine bone mineral density (LSBMD) should be investigated via DXA.

In addition, there was a strong positive correlation between the BHI-SDS and SSI-SDS, which was also observed in children and adolescents with type 1 diabetes mellitus [53]. The SSI can reliable estimate the mechanical strength of human forearm bones to handle bending and torsion [9]. According to the Utah paradigm of skeletal physiology, it is important to interpret the SSI along with the grip strength and the muscle cross-sectional area, because the strength of bones after birth relies strongly on the mechanical load of bone by muscle force [54]. In healthy children, bone strength continuously adapts to the increasing mechanical loads produced by muscle contraction during stature growth, ensuring that bone strength adapts to muscle strength [20]. In this study, we found a positive but not significant correlation between BHI-SDS and muscle cross-sectional area SDS as well as grip strength SDS. Though the methods showed good overall agreement, grip force showed a proportional bias, with increased differences in higher mean values, which limits the usefulness of grip strength. However, these findings may suggest that bone strength in adolescents with endocrinopathies adapts adequately to muscle size and strength. In addition to that we could show a low fracture prevalence (3.7%) throughout the whole cohort compared to reference data of middle Europe (3–5%) [55].

The gold standard for assessing bone health in adolescents and children remains the subject of ongoing debates. A direct comparison of BHI and pQCT measurements is essentially comparing two different methods that look at different indicators in different areas of the bones. Three-dimensional bone density has been analysed by pQCT at two sites in the radius, distinguishing between cortical and trabecular bone architecture [9]. Useful SDS values for CA and height age are available [9]. The effective radiation of pQCT using the Stratec device is less than 1 µSv [10]. Adams et al postulated, that based on values in adults [56], one can estimate that pQCT radiation exposure is comparable to or slightly lower than for DXA [10]. pQCT is time-consuming and susceptible to motion artifacts, especially in young patients, and is not always available due to a lack of trained personnel or financial resources. In contrast, the BHI describes bone mass as a function of the cortical thickness of three metacarpals and their width and length [16]. This method is particularly advantageous in paediatric patients as it takes into account variations in height and specific SDS values established for both children and adults [16,18]. Furthermore, the effective radiation dose of a hand radiograph is very low, ranging from 0.10 to 0.12 μSv for children aged 10–15 years [57]. This is comparable to an internal examination of radiation doses in our facility, which showed an effective radiation dose of <0.1 µSv (Data not shown). Consequently, if the BHI is based on a radiograph taken to determine bone age, the BHI measurement is obtained at no additional radiation exposure or cost.

The relationship between bone mass and bone health risk is poorly understood, particularly as the BHI is performed on the metacarpals, which are a different site of common fractures. But other authors showed that hand maturation, including the metacarpals, can be used as an index for the rest of the skeleton´s development [58,59]. Therefore, it seems reasonable to hypothesize that the metacarpals can be an index for overall mineralization. All established methods have significant limitations and can only provide an approximate assessment of bone health, with the fracture prevalence probably the only true indicator of bone quality and bone strength [60].

The strengths of our study are the comprehensive analysis of the functional muscle–bone unit using established methods (pQCT and hand dynamometry) and comparing these results with BHI measurements in patients with chronic endocrine diseases at the time of transition from pediatric to adult healthcare. However, the cross-sectional design is a limitation. Another limitation is that it is not known whether the findings from the functional muscle–bone unit of the hand represents whole-body bone status. Additionally, we did not investigate trabecular bone mass using DXA. Finally, the number of patients in each group is small, and further prospective studies are required.

In conclusion, this study demonstrates for the first time the BHI assessed by BoneXpert^TM^ for different endocrine diseases and a good correlation with pQCT measurements in adolescents. Additionally, the BHI measured by BoneXpert^TM^ is an effective way to evaluate bone health that is widely available, automated, and involves low radiation exposure, making it suitable for regular patient care along with bone age assessment. The BHI and grip strength provide insight into the muscle–bone relationship and can be easily assessed in an outpatient setting. However, the BHI may only reflect certain aspects of bone quality, as there seems to be a systematic underestimation of certain parameters (BMD, BMC and CSA), but as a stable parameter with a coefficient of variation of 1.4% [33], in our opinion, it has the potential to estimate bone health in children and adolescents and may could be used as an effective screening tool. However further studies are needed.

## Supporting information

S1 Table Patient characteristics of the pQCT subgroup. CAH congenital adrenal hyperplasia; GHD isolated growth hormone deficiency; SGA small for gestational age; TS Turner syndrome; f female; m male; Data are given as mean ± standard deviation (SD). Age is given in median and IQR. Statistics by ANOVA: *p < 0.05 vs. CAH and UTS; + p < 0.05 vs. SGA. × p < 0.05 vs. CAH, SGA and GHD. Statistics by Kruskal-Wallis-Test: §p < 0.05 vs. CAH and UTS; %p < 0.05 vs. SGA.
(DOCX)

S2 File Data Table. All patient parameters; SDS standard deviation score.
(XLS)

S3 Table Linear regression analyses of the differences and means of all pQCT parameters compared with BHI-SDS. (DOCX)
