# Supplementary material for: Bone Health Index (BoneXpert) and parameters of peripheral quantitative computed tomography indicate overall adequate bone health in adolescents with chronic endocrine diseases at time of transition
Source: PLoS One. 2025 Dec 4;20(12):e0337842. doi: 10.1371/journal.pone.0337842 (PMC12677445; doi:10.1371/journal.pone.0337842)
Supplement: S1 Table — CAH congenital adrenal hyperplasia; GHD isolated growth hormone deficiency; SGA small for gestational age; TS Turner syndrome; f female; m male; Data are given as mean ± standard deviation (SD). Age is given in median and IQR. Statistics by ANOVA: *p < 0.05 vs. CAH and UTS; + p < 0.05 vs. SGA. × p < 0.05 vs. CAH, SGA and GHD. Statistics by Kruskal-Wallis-Test: §p < 0.05 vs. CAH and UTS; %p < 0.05 vs. SGA. (DOCX) [file pone.0337842.s001.docx]

**S1 Table:** Patient characteristics of the pQCT subgroup

| **Diagnosis** | **Total** | **CAH** | **GHD** | **SGA** | **TS** |
| --- | --- | --- | --- | --- | --- |
| **Biological sex (f/m)** | 38 (22/16) | 13 (6/7) | 12 (6/6) | 6 (3/3) | 7 (7/-) |
| **CA (years)** | 17.4  (16.2-20.3) | 18.9  (17.7-22.8)^%^ | 16.9  (15.9-19.8) | 15.4  (14.7-16.8)^§^ | 17.7  (16.7-20.7)^%^ |
| **BA (years)** | 16.4  (15.3-17.5) | 17.1  (15.8-17.7) | 16.4  (14.9-17.4) | 15.7  (14.4-17.2) | 16.4  (1543-17.7) |
| **Difference CA-BA** | 1.5  (-0.4-5.4) | 2.6^%^  (-0.2-6.3) | 0.9  (-0.8-3.7) | -0.6  (-0.9-0.1) | 3.4  (-0.2-5.6) |
| **Near Final Height (NFH) SDS** | -1.79  (-2.38- -0.62) | -0.86  (-2.28- -0.21) | -1.46  (-1.94- -0.29) | -2.14  (-2.83- -1.45) | -2.69  (-3.41- -1.11) |
| **Target Height (TH) SDS** | -0.53  (-1.59- -0.09) | -0.30^+^  (-1.06-0.06) | -0.65  (-1.50- -0.16) | -1.72*  (-1.88- -0.45) | -0.39^+^  (-1.08-0.88) |
| **Difference NFH - TH** | -0.77  (-1.74- -0.04) | -0.47  (-1.58- -0.01) | -0.24  (-1.25-0.59) | -0.66  (-1.45- -0.15) | -2.33^×^  (-3.25- -1.99) |
| **BMI SDS** | -0.08  (-1.16-1.24) | -0.05  (-0.46-1.44) | -0.37  (-1.88-0.56) | -0.18  (-1.59-1.36) | 0.92  (-0.26-2.04) |

*CAH* congenital adrenal hyperplasia; *GHD* isolated growth hormone deficiency; *SGA* small for gestational age; *TS* Turner syndrome; *f* female; *m* male; Data are given as mean ± standard deviation (SD). Age is given in median and IQR. Statistics by ANOVA: *p < 0.05 vs. CAH and UTS; ^+^p < 0.05 vs. SGA. ^×^ p < 0.05 vs. CAH, SGA and GHD. Statistics by Kruskal-Wallis-Test: ^§^p < 0.05 vs. CAH and UTS; ^%^p < 0.05 vs. SGA.
