# Supplementary material for: Bone Health Index (BoneXpert) and parameters of peripheral quantitative computed tomography indicate overall adequate bone health in adolescents with chronic endocrine diseases at time of transition
Source: PLoS One. 2025 Dec 4;20(12):e0337842. doi: 10.1371/journal.pone.0337842 (PMC12677445; doi:10.1371/journal.pone.0337842)
Supplement: S3 Table — (DOCX) [file pone.0337842.s003.docx]

**S3 Table. Linear regression analyses of the differences and means of all pQCT parameters compared with BHI-SDS**

| Parameter | Regression coefficient B | Significance **p** |
| --- | --- | --- |
| Total bone mineral  density_65%_ (SDS) | -0.090 | 0.784 |
| Cortical bone mineral  density_65%_ (SDS)  Bone mineral content_65%_ (SDS)  Total bone mineral  density_4%_ (SDS)  Trabecular bone mineral density_4%_ (SDS) | -0.391  0.192  -0.192  0.032 | 0.297  0.242  0.378  0.903 |
| Cortical thickness (SDS) | -0.031 | 0.867 |
| Total cross-sectional area (SDS) | -0.418 | 0.079 |
| Strength-Strain Index (SDS)  Muscle cross sectional area (SDS)  **Grip strength (SDS)** | 0.232  0.203  **0.962** | 0.319  0.369  **<0.001** |

Significant correlations are highlighted (bold).
